# Question Popularity Analysis and Prediction in Community Question Answering Services

**DOI:** 10.1371/journal.pone.0085236

**Published:** 2014-05-16

**Authors:** Ting Liu, Wei-Nan Zhang, Liujuan Cao, Yu Zhang

**Affiliations:** 1 Research Center for Social Computing and Information Retrieval, Harbin Institute of Technology, Harbin City, Heilongjiang, China; 2 Department of Computer Science, School of Information Science and Technology, Xiamen University, Xiamen City, Fujian, China; University of Warwick, United Kingdom

## Abstract

With the blooming of online social media applications, Community Question Answering (CQA) services have become one of the most important online resources for information and knowledge seekers. A large number of high quality question and answer pairs have been accumulated, which allow users to not only share their knowledge with others, but also interact with each other. Accordingly, volumes of efforts have been taken to explore the questions and answers retrieval in CQA services so as to help users to finding the similar questions or the right answers. However, to our knowledge, less attention has been paid so far to question popularity in CQA. Question popularity can reflect the attention and interest of users. Hence, predicting question popularity can better capture the users’ interest so as to improve the users’ experience. Meanwhile, it can also promote the development of the community. In this paper, we investigate the problem of predicting question popularity in CQA. We first explore the factors that have impact on question popularity by employing statistical analysis. We then propose a supervised machine learning approach to model these factors for question popularity prediction. The experimental results show that our proposed approach can effectively distinguish the popular questions from unpopular ones in the Yahoo! Answers question and answer repository.

## Introduction

Community Question Answering services have emerged as extremely popular alternatives for online information acquisition, such as Yahoo! Answers (http://answers.yahoo.com/), WikiAnswer (http://wiki.answers.com/) and Baidu Zhidao (http://zhidao.baidu.com/), etc. According to Google Trends (http://www.google.com/trends/), all the above three CQA services had more than 10 million searches and visits in 2011. Over time, a huge amount of question and answer (QA) pairs with high quality devoted by human intelligence has been accumulated as comprehensive knowledge bases. It greatly facilitates users to seek precise information by obtaining correct answers directly, rather than to painstakingly browse through large ranked lists of results.

Moreover, as a social media service, CQA provide a platform for users to not only ask and answer questions, but also share their social behaviors with each other, such as voting for high quality content, representing opinions to existing answers and showing their interests on specific questions etc. Series of studies are taken to investigate user vote, opinion and other social information to identify or predict the answer quality [1]–[6], question similarity [7]–[9] and question quality [10] in CQA services. However, as for question popularity, few literatures have been documented. In fact, CQA questions tend to attract user attention so as to obtain more answers and comments. Table 1 shows the example questions belonging to the same category (“Cooking & Recipes”) and submitted in the same year (2008) in Yahoo! Answers.

**Table 1:** User attention distribution on different questions in Yahoo! Answers.

Question	# of interesting tags	# of Answers
*Q1*: Besides lasagna, what are some good family-friendly meals that you make ahead of time?	359	601
*Q2*: What are some great holiday cookie recipes that can be made quickly?	189	225
*Q3*: How do you cook baked chicken with boxed stuffing?	0	0

For a given question, “# of interesting tags” indicates the number of users who are interested in the question while “# of answers” represents the number of answer attempts. From Table 1, we can observe that *Q1* and *Q2* acquire hundreds of interesting tags and answers while *Q3* fails to obtain any interesting tags and answers. That indicates varied degrees of question popularity.

The significance of exploring question popularity in CQA is three-fold:

- Question popularity reflects users’ interests. Users lean towards paying more attention to the questions that they are interested in. Therefore, crowd interests indicate question popularity.
- Question popularity improves answering efficiency. It is observed that popular questions can attract more answering attempts and discussions from users. Hence, popular questions can be resolved more efficiently.
- Question popularity facilitates information and knowledge acquisition. As popular question and answer pairs are more informative and knowledgeable, it will become a high quality resource not only for CQA services, but also for search engines.

However, modeling question popularity is a non-trivial task. First, there are many factors that affect question popularity. We classify these factors into three categories: (1) content related factors; (2) social behavior factors; and (3) user profile factors. Hence, **how to find factors affecting question popularity** becomes one problem. Second, series of studies focus on predicting popularity of news [11], events [12], views [13], social images [14], [15], marketing messages [16], landmarks [17], [18] and question quality [10] etc. However, few literatures have so far been documented on predicting question popularity in CQA. Therefore, **how to effectively model the factors for question popularity prediction** is another problem.

Addressing these problems, we apply statistical analyses to explore factors affecting the question popularity. Based on the results of factor analyses, we effectively predict question popularity using a supervised machine learning approach.

## Related Work

Recently, predicting the popularity of online content, especially the User Generated Content (UGC), becomes a hot research topic. Szabo and Huberman [13] proposed a log-linear model to predict the popularity of online content in Youtube (www.youtube.com) and Digg (http://digg.com/) data. They modeled the users’ votes and views of the online content to predict the dynamic of individual submissions. Richardson et al. [19] utilized a probabilistic model to capture the position information, the viewed information and the clicked on information. They then used the above information to predict the click-through rate of the new advertisements. Lerman [20] employed a mathematical model to describe the dynamics of the collaborative rating of the news quality by modeling the users social influence through their social networks. The author then presented that the proposed model can effectively predict the user rating behaviors in Digg. In 2010, Lerman and Hogg [11] captured the social dynamic information in Digg and used a linear model to predict the popularity of news by modeling the user votes. Despite the success of the previous efforts, literatures regarding the multiple dimensions of the social media for the content popularity prediction are still sparse.

With the blooming of social media applications, popularity prediction on Twitter (https://twitter.com/) attracts more and more attention of researchers. Yang and Counts [21] utilized a diffusion network to measure the topic propagation in Twitter. They then used the proposed model to predict the speed, scale and range of diffusion information in Twitter. Hong et al. [22] investigated the problem of predicting the popularity of messages in Twitter. They captured the user behavior feature (retweet) and content feature (TF-IDF) to predict the future number of retweets and sheds. Gupta et al. [12] employed 4 regression models and 1 classification model to model the popularity features, ratio features, social features and out-of-Twitter popularity features for events popularity prediction. They then proposed a hybrid model to effectively capture the features of the four categories and obtain better performance. Although there is an amount of existing efforts on content popularity prediction in Twitter, the features they utilized are too specific to transfer to other social media applications.

In this study, we focus on a new research topic, namely question popularity prediction in CQA services. Inspired by the above studies, we proposed a supervised machine learning approach to predict question popularity by modeling content related, user behavior and user profile features.

## Factors Affecting Question Popularity

**Figure 1:**
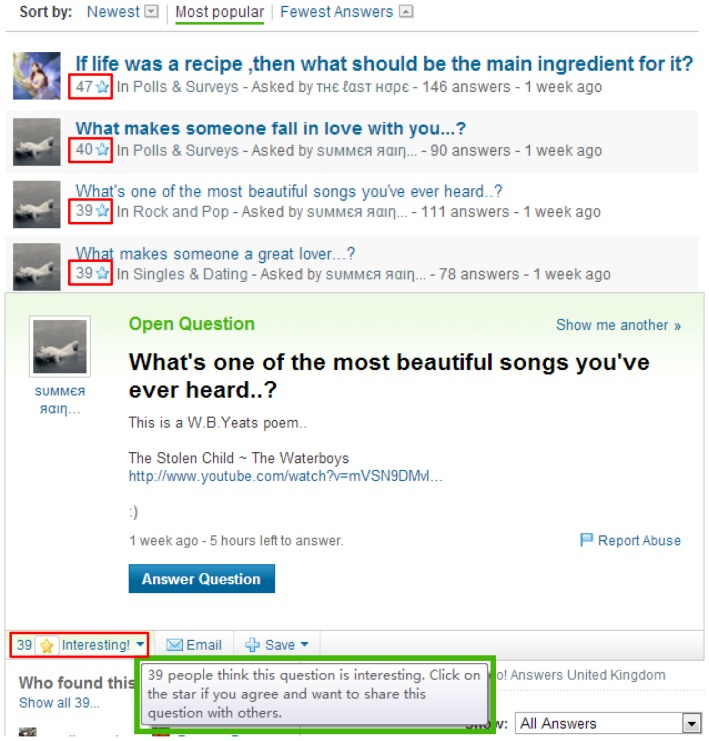
Example of user interesting tags in Yahoo! Answers questions. The content in the red box represents the number of interesting tags labeled by users. That is a rational and effective indicator of question popularity.

In this section, we will utilize statistical analyses to explore the factors affecting question popularity. We collected a total number of 199,765 questions from the Yahoo! Answers using the Yahoo! Answers API. We randomly sampled 50,000 questions for testing. The remaining questions are used for the statistical analysis. As previously described, crowd interests can indicate question popularity. Hence, we can employ the number of user interesting tags as the indicator of question popularity. Here, the interesting tag is a button which is labeled with a star in Yahoo! Answers online service. If the users are interested in the current question, they can click on the star button. Hence, the number of interesting tags equals to the number of interested users. Figure 1 shows the example of user interesting tags in Yahoo! Answers questions.

Next, we analyze the three categories of factors in details.

### Content Related Factors

Content related factors include question category and question content quality. We make two assumptions.

1 The distributions of question popularity are different among question categories.
2 Questions with lower content quality usually fail to attract users’ attention.

#### Question category

Our collected CQA data cover 1116 categories in Yahoo! Answers. However, the distribution of question quantity among these categories are unbalanced as well as the distribution of question popularity. For example, in the category *“Rock and Pop”*, we obtain a total number of 983 popular questions. The sum of these questions’ popularity value equals to 27220. While, in the category *“Australian Rules”*, we collect a comparative quantity (957) of popular questions, the sum of the questions’ popularity value only equals to 3669. For the consistence of popular question definition with Yahoo! Answers, we utilize the number of user interesting tags, for a given question, as the popularity value.

**Figure 2:**
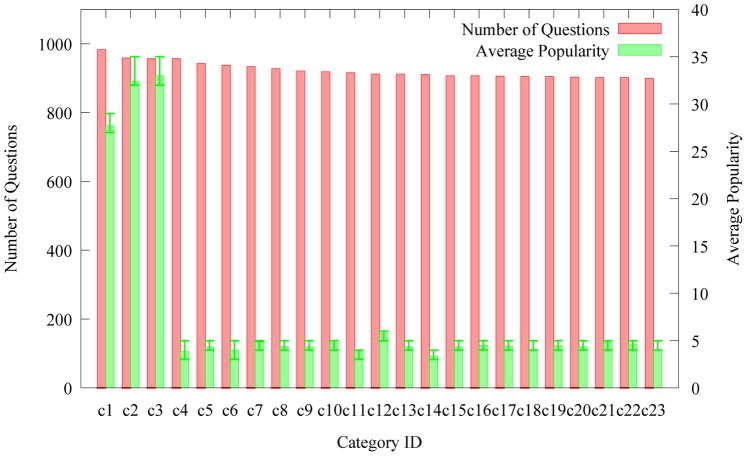
The distributions of question quantity in a category and average popularity of the category on the subset of Yahoo! Answers taxonomy.

To capture the differences of question popularity distribution among different categories, we first select 23 categories from our collected data. Each of them contains comparative quantity of questions (900–983). We define category popularity as the sum of all the questions’ popularity in a specific category. Then, we introduce the average popularity measure. In a category, it can be calculated by the quotient of the category popularity and the total number of questions. Therefore, we can eliminate the popularity bias on question quantity. Figure 2 shows the distributions of average question popularity and question quantity on 23 categories.

From Figure 2, we can see that the average popularity values of the categories “c1 (Rock and Pop)”, “c2 (Jokes & Riddles)” and “c3 (Religion & Spirituality)” are higher than those of the categories “c21 (Drama)”, “c22 (Royalty)” and “c23 (Comedy)”. We also notice that no association can be observed between number of popular questions in category and popularity of the category. For example, the categories “c5 (Alternative Medicine)” and “c6 (R&B & Soul)” contain 943 and 938 popular questions respectively.

#### Question content quality

**Figure 3:**
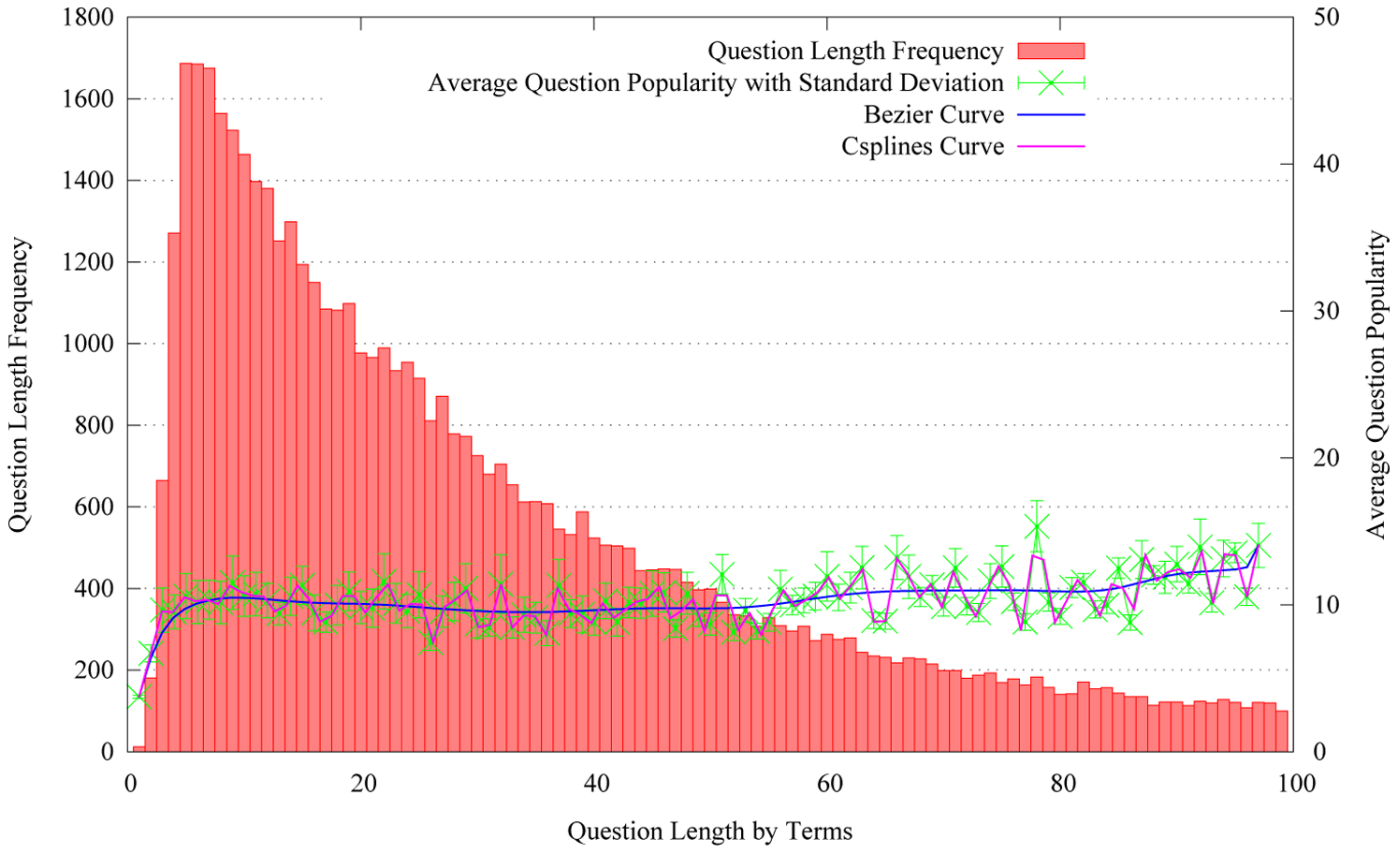
The distribution of average question popularity on question length.

Inspired by previous work on content quality estimation [2], [23], we utilize question length to measure the question content quality. Here, question length represents the number of unique tokens or terms for each question after removing stop words. As questions in Yahoo! Answers have two components, question title and question description, we consider the two components together for counting question length. Meanwhile, we also count the frequency of the questions by their length. Hence, the average question popularity in Figure 3 equals to the sum of the question popularity divided by the total number of questions of the specific length. For example, there are 1,700 questions which have the same question length 10. And the sum of the popularity for the 1,700 questions equals to 17,000. Hence, the average question popularity in length 10 equals to 10. Figure 3 indicates the average question popularity distribution on question length.

From Figure 3, to see the green line with error bars, we can observe that the average question popularity sharply increases when the question length varies from 1 to 10. Then, it is stable when the range of the question length is from 10 to 55. After that, it continuously increases. Meanwhile, we also notice the length of the majority of questions belong to the range of 1 to 20. We can observe that the average question popularity varies consistently with the length of questions.

### Social Behavior Factors

After exploiting the relation between question content factors and question popularity, we consider the social behavior factors. For CQA questions, we can capture their question social quality [10], the number of answers, the submitted time and the time they were responded for the first time. Here, we make three assumptions as follows.

3 The number of answers can be a indicator for the question popularity. The more answers a question holds, the more popular it is.
4 We assume that the popularity of the questions can be cumulated by the lapse of time.
5 Meanwhile, we assume that the popular questions can be answered more quickly. Hence, the response time can be a indicator for question popularity.

#### Number of answers

**Figure 4:**
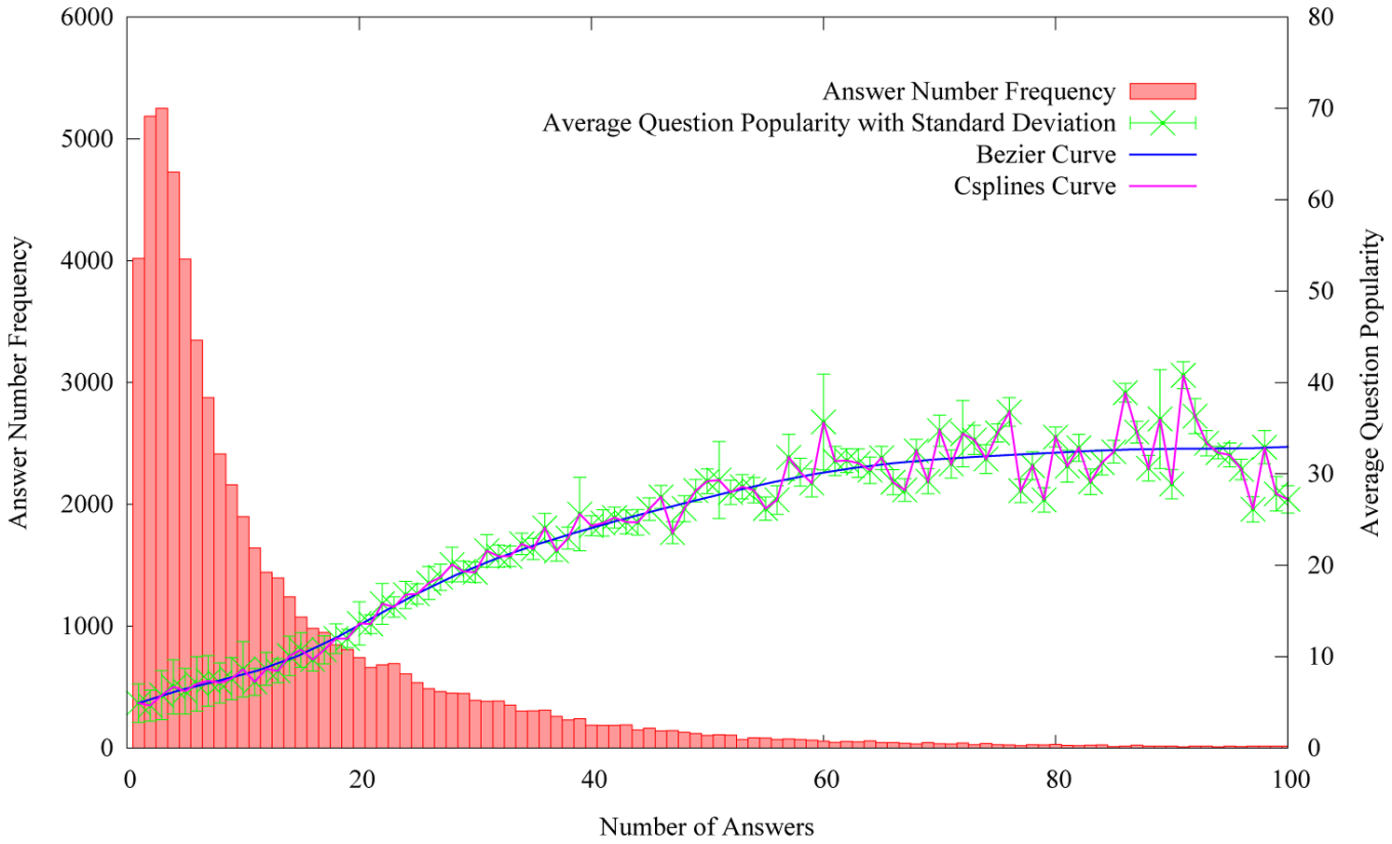
The distribution of average question popularity on the number of answers.

As Table 1 shows, the more popular questions may obtain more answers. Therefore, we design to explore the relation between question popularity and the number of answers. Here, the average question popularity in Figure 4 equals to the sum of the question popularity divided by the number of questions that have a specific number of answers. For example, there are 2,100 questions, each of which has 10 answers. And their sum of popularity equals to 18,900. Hence the average question popularity equals to 9. Figure 4 shows the distribution of average question popularity on the number of answers by statistics.

Figure 4 shows the average popularity of questions, which obtain less than 10 answers, is lower than 10. It means that most of user interests are attracted by a small number of questions. To see the green line with error bars, we can observe that the average question popularity increases consistently with the growing number of answers. It reveals that popular questions can attract more answer attempts by users.

#### Question submission time

**Figure 5:**
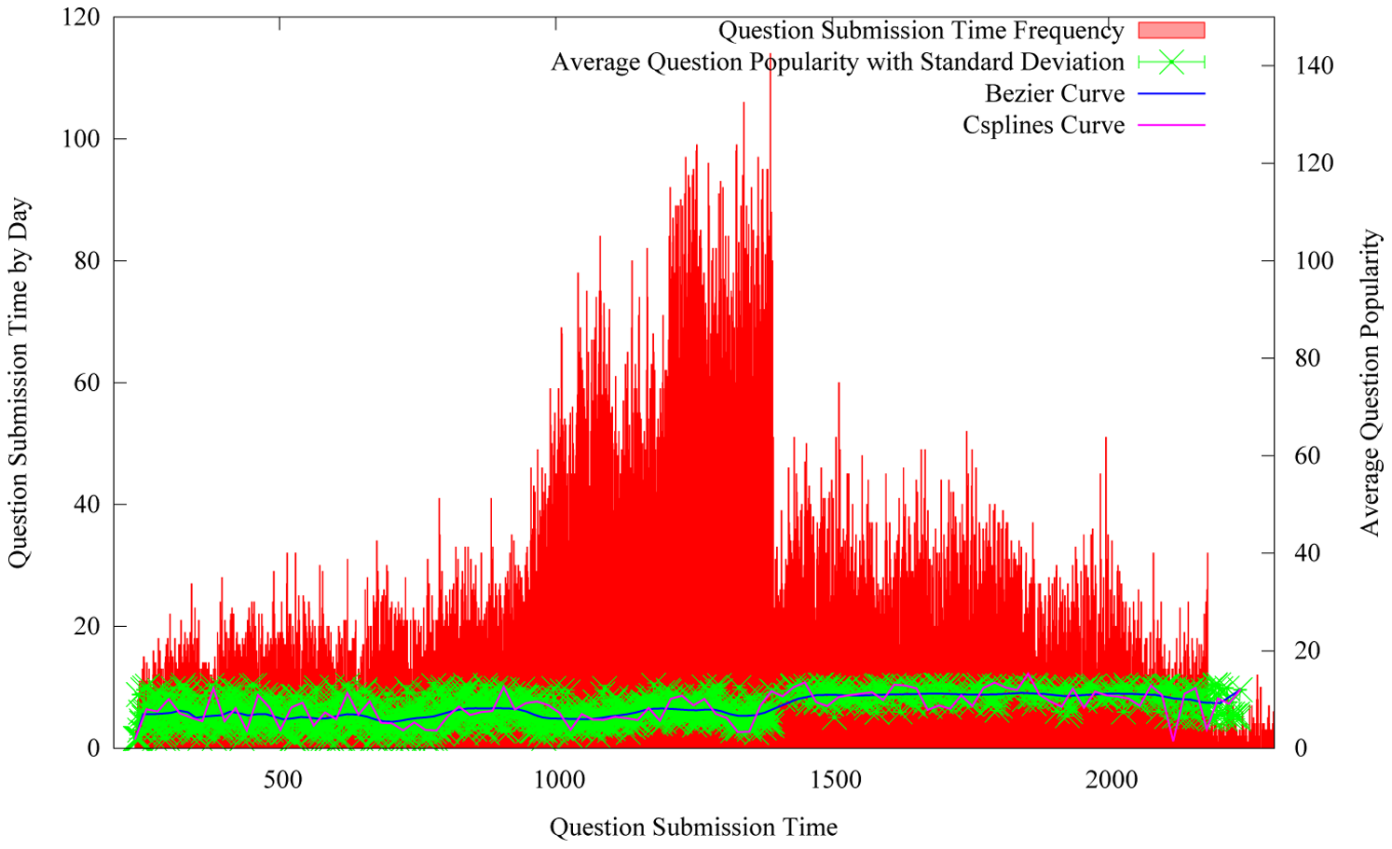
The variation of average question popularity with the growing of the question submission time. The time unit for statistic is one day.

As we assumed, question popularity can be accumulated by time. It seems to be obvious. However, what we actually want to verify is that popular questions can continuously attract users attention. Hence, we first capture the relation between the question submission time and the average question popularity. The time point for the statistic of submission time is Oct 1, 2012. Here, the average question popularity in Figure 5 equals to the sum of the question popularity divided by the number of questions that have a specific “age”. For example, there are 100 questions which have the same “age” of 20 hours. The sum of the question popularity equals to 4,000. Hence the average question popularity equals to 40. Figure 5 shows the variation of the average question popularity with the growing of the submission time.

Figure 5 shows the trend of the average question popularity increases with the increase of the question submission time. When the question submission time is larger than 2200, the average question popularity sharply increases. However, to see the red boxes, only a small number of questions obtain the higher average question popularity. Summarized, we can observe that the average question popularity increases stably with the growing of question submission time.

#### Question response time

**Figure 6:**
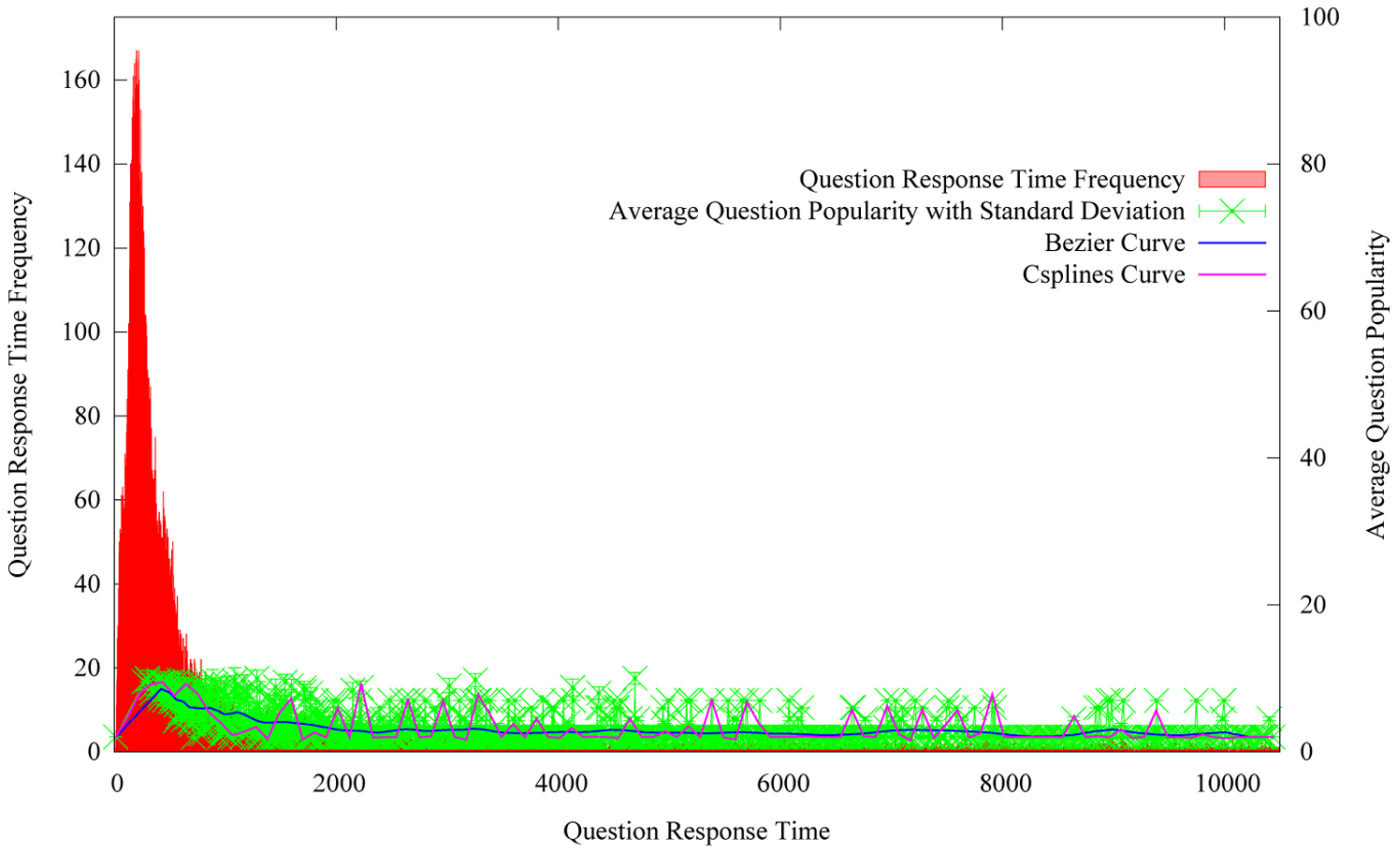
The variation of the average question popularity with the growing of the question response time.

Another time related factor is the question response time. Here, the response time represents the interval between the question was submitted and the question was answered for the first time. We try to explore the relation between the average question popularity and the question response time. Here, the average question popularity equals to the sum of the question popularity divided by the number of questions which have the same response time. For example, there are 20 questions which have the same response time. The sum of the question popularity equals to 160. Hence the average question popularity equals to 8. Figure 6 represents the variation of the average question popularity with the growing of the question response time.

Figure 6 shows that the average question popularity decreases with the growing of the question response time. It indicates that the shorter the response time, the more popular the corresponding questions are. It also verifies that the popular questions can attract user attention more quickly. Popular questions are more likely to be resolved efficiently.

### User Profile Factors

Another important information in User Generated Content (UGC) and its applications, such as CQA services, is the user profile. In this study, we capture two aspects of user profiles. One is the asker’s level. The other is the asker’s popularity. We make the following assumptions.

6 Here, we assume that the asker level is highly related to the question popularity. If the current question is asked by the user with higher level, it is more likely to be a popular question.
7 Similar to the last assumption, we consider that if the current question is asked by the users with more popularity, it is more likely to be a popular question.

#### Asker level

**Figure 7:**
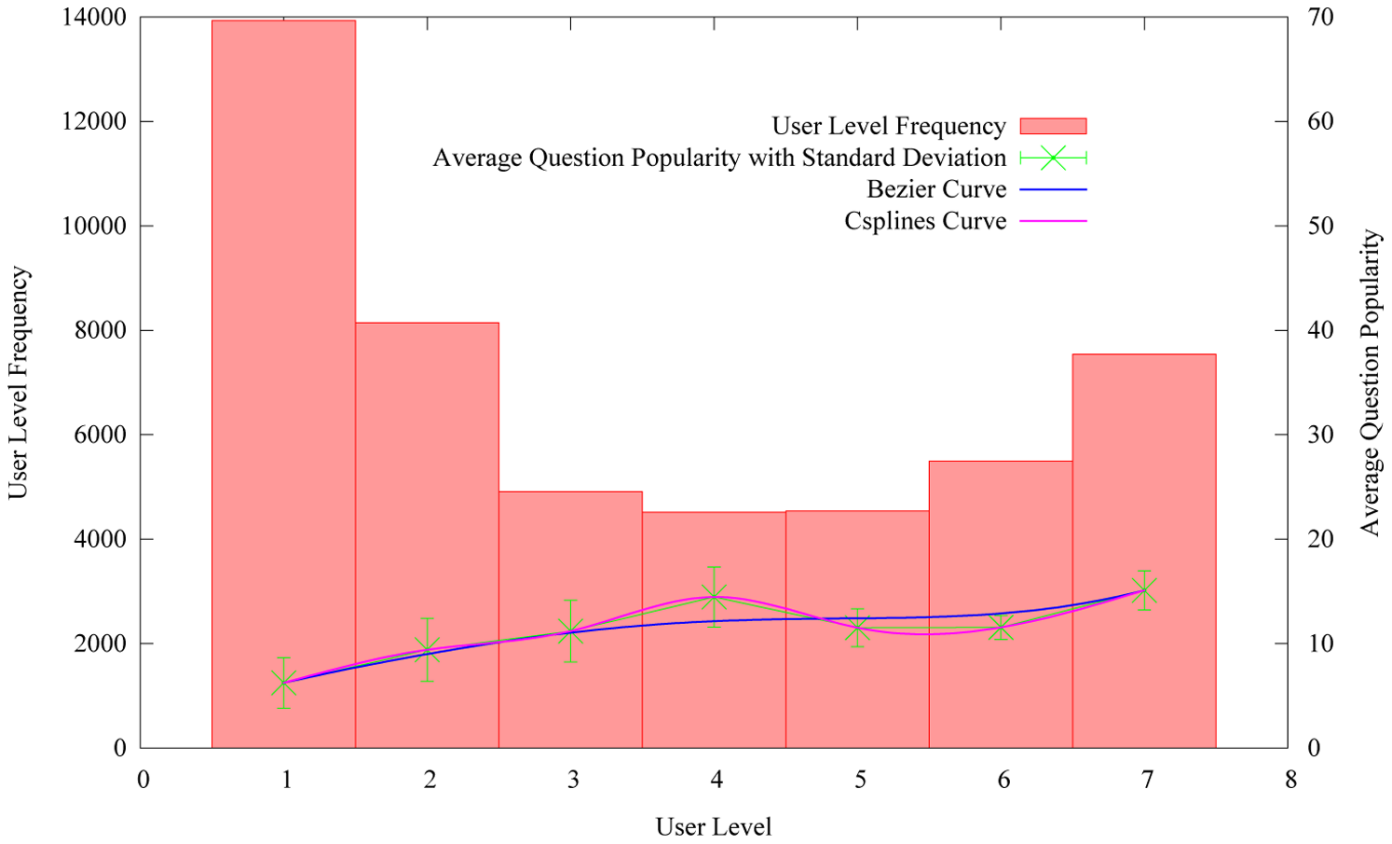
The distribution of average question popularity on the user level.

We first capture the relation between the asker level and the average question popularity. The value of asker level can be calculated by the times of user interactive behaviors when using CQA services, such as asking new questions, answering other questions, voting for best answers and giving comments for other answers, etc. The more frequent user interactive behaviors are, the higher the user level is. Here, the average question popularity equals to the sum of the question popularity divided by the number of users who have the same user level. For example, there are 8,000 users whose user level equals to 2. The sum of the question popularity which obtained by the users equals to 72,000. Hence the average popularity equals to 9. Figure 7 shows the values of average question popularity varying with the user level. Here, in Figure 7, user represents the asker.

Figure 7, shows that the average question popularity consistently increases with the user level. It verifies the assumption that the questions submitted by higher level users are more popular than those submitted by lower level users. It illustrates that the user level is a good indicator for question popularity. It also indicates that the users with higher level usually tend to publish popular information or topics.

#### Asker popularity

The other important information in the user profile is the asker’s popularity. In this study, the asker’s popularity equals to the sum of the questions popularity asked by him or her. It is obvious that the more popular the asker’s questions are, the higher the asker’s popularity is.

**Figure 8:**
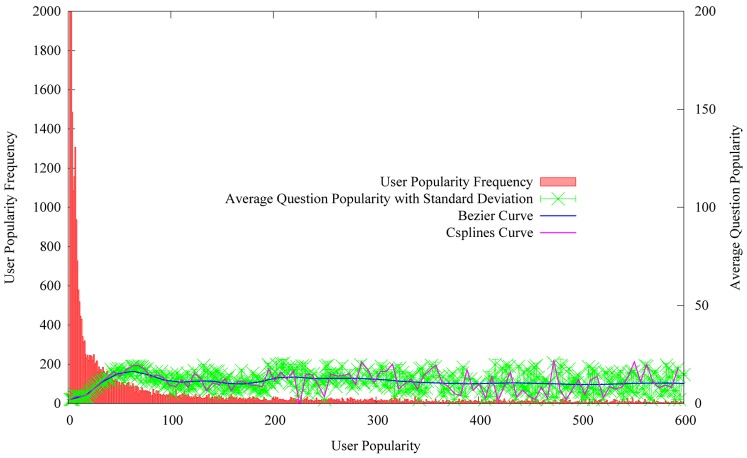
The distribution of average question popularity on the user popularity.

Unlike the asker level factor which is calculated by the behaviors of the askers themselves, the asker popularity factor is obtained by other users’ actions, such as tagging the askers’ questions as interesting. Hence, the asker popularity factor can provide the prior knowledge for question popularity prediction. Thus, it indirectly reflects the popularity submitted by the asker. Here, the average question popularity equals to the sum of the popularity divided by the number of users who have the same user popularity. For example, there are 110 users who are historically obtained 50 popularity. The sum of the question popularity equals to 1,650. Hence, the average question popularity equals to 15. Figure 8 shows the variation of average question popularity with the growing of user popularity.

From Figure 8, to see the green line with error bars, we can observe that the average question popularity consistently increases with the user popularity. To see the red boxes, we can conclude that the popularity of voluminous users is less than 10. While, the small number of users with higher average popularity can attract most of user interests. Hence, we can speculate that the users with higher popularity are more likely to submit popular questions.

## Question Popularity Prediction

Based on the above factor analysis, in this section, we will focus on how to effectively model these factors for the question popularity prediction task. Here, we use 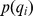 to represent the popularity of the given question 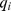. We then make the following assumptions:

*Assumption A.*

Each question 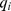 can be assigned to one of the mutual exclusive classes: **PC** (popular questions class) or **NPC** (non-popular questions class).

*Assumption B.*

There exists a global function 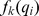, which indicates the “confidence” that the question 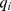 belongs to the class **PC**.

Instead of directly estimating 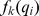, we employ a normalized variant of 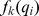 to derive the estimation:
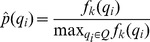
(1)


where 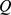 represents the whole candidate question set of which the popularity is to be predicted.

Inspired by previous efforts on the online content popularity prediction [12], [16], [24] to deduce the global function 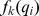, we utilize a supervised machine learning approach, which can seamlessly adopt the factors described in the section of Factors Affecting Question Popularity, to predict the question popularity.

Formally, we can obtain a training set of labeled instances:
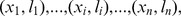



where 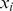 represents the feature vector of the question 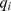, and 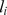 is a binary label which indicates whether 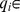 **PC**. Given the training set, we want to find a ranking function of the form 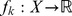, where 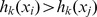 indicates that the question 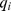 has the higher confidence to belonging to the class **PC** than the question 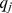. After the learning process, we can estimate the 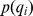 by directly using the global function 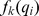 as shown in Equation (1).

Next, we will introduce the features used for the question popularity prediction task. These features are deduced from the factors which we have analyzed in the section of Factors Affecting Question Popularity. Table 2 gives the summary of the feature set used for question popularity prediction. Meanwhile, we will detail these features as follows:

**Table 2:** A summary of the features used in the question popularity prediction task.

Feature Category	Feature Name	Feature Description
Content Related	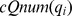	the number of the question in the category that 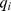 belongs to.
	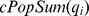	the sum of the question popularity in the category that 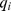 belongs to.
	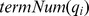	the number of unique terms in the question 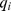 .
Social Behavior	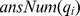	the number of answer attempts for the question 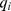 .
	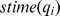	the time span from statistical deadline to the time that the question 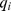 being submitted.
	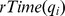	the time span between the question submission time and the time that the question 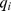 obtain its first answer.
User Profile	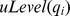	the level of the asker who submits the question 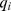 .
	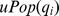	the total popularity of the asker who submits the question 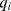 .

## Experimental Results

### Data Set

We collect a total number of 199,765 questions by the end of Oct 1, 2012 from Yahoo! Answers using the Yahoo! Answers API (http://developer.yahoo.com/answers/). It covers a range of popular topics, including 1116 subcategories. For each question, we extracted the content information (the texts of subject, content and answers and the categories), social behavior information (the number of answers, the submission time and the response time) and the user profile information (asker levels and asker popularity) as features from the returned questions by the API. For the question popularity prediction task, we randomly select 50,000 questions from our collected data set. After removing the incomplete questions (some of the collected questions lost the information of user level or user popularity), we obtained a total of 48,976 questions for training and testing, which covers 736 subcategories. Table 3 shows the statistical information of our training and testing data set.

**Table 3:** The statistical information of the training and testing data set.

**Total number of questions**	48976
**Number of covering subcategories**	736
**The range of question number in subcategories**	1–983
**The range of question popularity**	2–658

To obtain the ground truth of the question popularity prediction task, we normalize the popularity of each question, which is indicated by the number of the user labeled interesting tags, by dividing the maximum popularity value around the whole training and testing data set. We thus map the user labeled question popularity value into the interval of 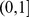. We then set the threshold 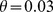. The questions which their popularity are larger than 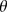 are labeled as popular questions, vice versa. Finally, we obtain a total of 19,176 popular questions and 29,800 unpopular questions respectively. We released all the raw questions and the transformed feature representations used in our experiments in https://www.dropbox.com/s/zszf75ep8uy1hdw/YahooAnswerQA.sql and http://ir.hit.edu.cn/jzhang/share/YahooAnswerQA.sql, so that other researchers can easily use the data to reimplement our approaches without the feature extraction step.

### Question Popularity Prediction Results

To assess the effectiveness of the supervised machine learning approach on the question popularity prediction task, we employ the meta-classifier *AdaBoost.M1* with *J48 decision trees* as the base learners [25]. AdaBoost.M1 was shown to be more effective than the individual base learners [25], [26], such as C4.5 decision tree and Naive Bayes classifiers etc. Our empirical comparison of these classifiers also draw the same conclusion. In our experiments, all the prediction approaches and results analysis are performed by using the algorithms implemented in Weka [27], a collection of machine learning algorithms for data mining tasks (One can find Weka in http://www.cs.waikato.ac.nz/ml/weka/).

Next, we examined the question prediction results by using our proposed supervised machine learning approach. As presented in Table 2, we propose three categories of features, including the content related features, the social behavior features and the user profile features. We then set 7 comparison systems to assess the effectiveness of the above categories of features.

For each of the 7 systems, we utilized a 10-fold cross-validation and repeated 10 times to ensure the reliability of our results. For each system, the classifier was run 100 times. We thus obtain a total of 100 groups of prediction results. For each running, the sizes of training and testing set are 44078 and 4898 respectively. The final results of each system come from the average of the corresponding 100 groups of results. We then introduce the 7 systems as follows:

- **CF, SF, UF**: The systems which are only trained by using the content related features, the social behavior features and the user profile features respectively.
- **CF+SF, CF+UF, SF+UF**: The systems which are trained by using the combinations of the content related features and the social behavior features, the content related features and the user profile features and the social behavior features and the user profile features respectively.
- **CF+SF+UF**: Our proposed method which combines all of the content related features, the social behavior features and the user profile features.

Here, the CF and UF related approaches are prediction models. In the prediction models, only the content features and the user profiles are used to predict the question popularity. While the SF related approaches are classification models as they utilize the interactions between askers and answerers. Hence, the CF and UF based models can be employed to predict question popularity in the realtime applications.

The previous 6 systems are setting as the baseline systems. For the evaluation, we use the metric of 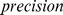, 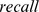 and 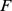-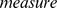. Table 4 shows the experimental results of the above 7 systems on the question popularity prediction task.

**Table 4:** Experimental results of the question popularity prediction.

Models (%)	CF	SF	UF	CF+SF	CF+UF	SF+UF	CF+SF+UF
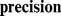 **(p)**	94.88	92.76	72.73	97.53	93.77	94.63	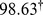
% **p** improvements over							
CF	N/A	N/A	N/A	+2.79	N/A	N/A	+3.95
SF	+2.29	N/A	N/A	+5.14	+1.09	+2.02	+6.33
UF	+30.46	+27.54	N/A	+34.10	+28.93	+30.11	***+35.61***
CF+SF	N/A	N/A	N/A	N/A	N/A	N/A	+1.13
CF+UF	+1.18	N/A	N/A	+4.0	N/A	+0.92	+5.18
SF+UF	+0.3	N/A	N/A	+3.06	N/A	N/A	+4.23
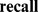 **(r)**	87.61	90.14	80.67	97.32	90.75	93.23	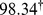
**F**- 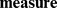 **(F)**	91.10	91.43	76.49	97.42	92.37	93.92	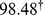

† indicates that the results of our proposed method are statistical significance over all of the 6 baselines (within 0.95 confidence interval using the paired *t*-test). The results of our approach are in bold.

From Table 4, we can observe that:

1) By comparing the results of CF, SF and UF, we found that SF received the best performance in 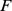-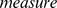. It demonstrates that the social behaviors are the most important features for the question popularity prediction task. This is because that the question popularity is calculated by the number of user interesting tags, which is essentially a kind of social behavior. While the other two categories of features indirectly reflect the question popularity from the content and user profile aspects respectively.

Meanwhile, we notice that only utilizing the user profile features (UF) cannot obtain better performance. This may be because that most of the user profiles are not distinguishable with each other in CQA services. From Figure 8, we can observe that only a few number of users have the extremely high popularity.

2) To see the 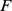-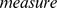 results, we can conclude that each of the CF+SF, CF+UF and SF+UF can obtain better performance than the CF, SF and UF respectively. It illustrates that each of the single category features can benefit by combining other features. Moreover, the performance of the CF+SF+UF further leads the same conclusion. This is because that even the question popularity is highly related with each of the above features, it cannot be effectively modeled by any of the single category features. Meanwhile, the users’ interest in a given question essentially depends on the mutual reinforcement of the question content itself, the social behavior of other users and the asker’s expertise. Hence, the CF+SF+UF can obtain the best performance than the other systems on the question popularity prediction task. In fact, in the real world applications of the question popularity prediction, the CF and UF features are more effective than SF. This is because the newly asked questions may fail to accumulate the user reactions.

We also noted that CF and/or UF is used to predicting the future popularity of the question while using SF is not really a prediction (because it uses the amount of reaction to the popularity of the question as features). It is natural that SF performs best (because it is in some sense tautological). We can also observe that it is very interesting that CF performs almost as good as SF. This is because the 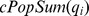 and 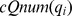 features can be seen as the prior knowledge which is highly related to the question popularity. And they contribute more than other features (to see the Table 5).

**Table 5:** Experimental results of the features analysis.

	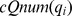	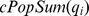	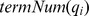	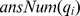	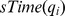	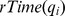	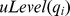	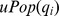
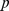	−0.52	−2.23	−0.03	−0.12	−0.04	+0.06	−**4.46**	−0.05
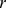	−0.52	−2.59	+0.09	−0.16	+0.02	−0.16	−**5.48**	+0.07
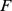	−0.52	−2.41	+0.03	−0.14	−0.01	−0.05	−**4.98**	+0.01
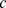	−**0.59**	**0.54**	0.11	**0.33**	0.10	−0.22	0.23	0.19

Each column represents the performance changes in percentage by removing the corresponding feature. “−” and “+” indicate the decrease and increase of the overall performance respectively. Features that contribute most for the question popularity prediction are marked in bold.

**Figure 9:**
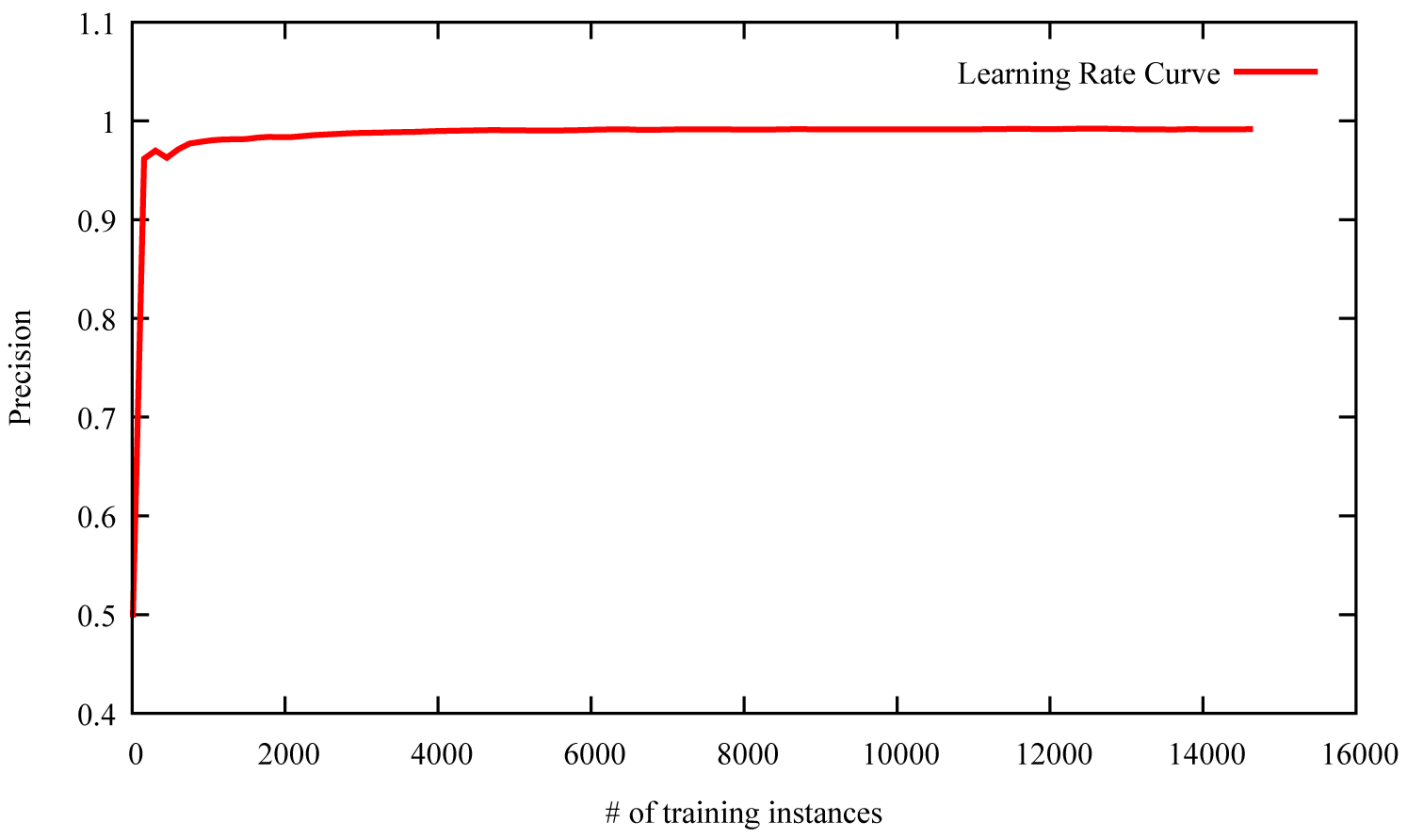
The learning rate of our proposed question popularity prediction approach.

We then examine how the number of training instances influences the question popularity prediction results. We randomly select 16,000 instances from our training and testing data set. A 10-fold cross-validation is utilized to ensure the reliability of the results. The final results come from the average results of all the 10 folds. Figure 9 shows the learning rate curve of our proposed question popularity prediction approach.

From Figure 9, we can observe that the precision of the question popularity prediction sharply increases with the growing of training instances when the number of training instances is smaller than 1000. It then tends to be stable when the number of training instances are larger than 1000. The curve demonstrates that the large amount of the training data is not necessarily needed for our proposed approach to achieve better performance. Hence, it is easy to transfer to other UGC popularity prediction tasks, especially to the tasks that have no sufficient labeling data.

### Feature Analysis

To further analyze the influence of our proposed features on the performance of the question popularity prediction task. In this section, we will assess the above (refer back to Table 2) features one by one. We repeat the question popularity prediction experiments using AdaBoost.M1 by varying the features. In each iteration, we remove only one single feature from the whole feature set. Here, we assume that these features are independent to each other. While the decrease in performance represents the contribution of the removed feature to the overall performance. Table 5 reports the results of the feature analysis experiments.

Here, 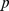,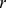,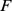 and 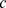 represent the precision, recall, F-score and the Pearson correlation coefficient values respectively.

From Table 5, we can observe that feature contribution to the overall performance is mainly consistent in different evaluation metrics. For the 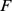-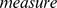, we can see that the 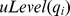 feature contributes most, followed by the 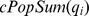 and 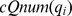 features. This demonstrates that user expertise is the most important feature for the question popularity prediction task. Meanwhile, the distribution of the question popularity and the number of questions on categories can also reflect the users’ attention and interest.

While, the 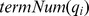 and 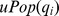 features have little negative impact on the overall performance. It illustrates that both of the 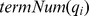 and 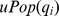 features are ambiguous to distinguish the popular questions from unpopular ones. The other features contribute more or less to the question popularity prediction task.

Meanwhile, we also check the Pearson correlation coefficient [28] of the features with the question popularity in Table 5. From Table 5, we can see that the 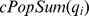 and 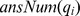 features are highly and positively correlated with question popularity. While the 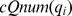 feature is highly and negatively correlated with question popularity. And the other features are also weakly correlated with question popularity. The p-value is less than 0.01 in the testing data for all the coefficient experiments by two-tailed test.

### Misclassification Case Analysis

In a posterior analysis, we find that there are mainly two kinds of misclassification cases.

First, the question content is not always a good indicator for the question popularity prediction. E.g., for the question “How much does it cost to make a penny?”, after removing the stop words, there are only 4 terms left. According to the Figure 3, the questions with their length equals to 4 are in the ambiguous interval for the identification of popular question. Meanwhile, the submission time is September 6, 2010 which is also in the interim interval in distinguishing popular and unpopular questions. Hence, it is wrongly classified as a unpopular question, while its popularity equals to 0.44.

Second, more answers not always means more popular of the corresponding questions. E.g., for the question “When did the hostilities between the English and Irish begin?”, it has a total of 25 answer attempts. However, its response time equals to 154 seconds which is in the boundary interval as shown in Figure 6. Meanwhile, its asker’s level equals to 1, but its asker’s popularity equals to 82, which is not quite consistent for the question popularity prediction. Hence, it is misclassified as a popular question, while its popularity is 0.03.

### Limitations of Our Proposed Approach

In this section, we will analyze the possible limitations of the relations between their featuring way and the features we used for question popularity prediction. On the one hand, the questions in Yahoo! Answers are classified into categories. And users have different interests in different topics and domains. Hence, the way of classifying questions may guide the way of users browsing the questions. Further it may affect the users scoring question popularity. On the other hand, Yahoo! Answers exhibit the questions partially according to the question submission time. Hence, it may lead the users focusing on new submitted questions. Further it may also affect the users scoring question popularity. Meanwhile, user level can reflect the attention of users. While, question popularity can reflect the users focus and interests. Hence, we can assume that popular users may lead to popular questions.

## Conclusion and Future Work

In this study, we investigated the problem of predicting question popularity in CQA services. We first utilized the statistical analysis to explore the factors that had the impact on question popularity prediction task. We then proposed a supervised machine learning approach to model the effective factors. Experimental results showed that our proposed approach obtained better performance while integrating all of the explored factors. By analyzing the learning rate curve, we deduced that our approach can easily transfer to other popularity prediction tasks with lower human labeled data.

In the future, we plan to exploring more content related features and the features from other social media. This is because that content related features perform better than the other features in our experiments. However, in this study, we only employ the category and question length features. We will capture more content features, such as language modeling feature and the similarity between question title and description feature.
